# The natural course of unruptured intracranial aneurysms in a Chinese cohort: protocol of a multi-center registration study in CIAP

**DOI:** 10.1186/s12967-019-2092-z

**Published:** 2019-10-22

**Authors:** Haixiao Liu, Wei Guo, Sishi Xiang, Peng Hu, Feifei Sun, Junmei Gao, Xiaoyang Zhang, Ping Wang, Wenting Jing, Lei Zhang, Xinjian Yang, Chuanzhi Duan, Min He, Hongqi Zhang, Yan Qu

**Affiliations:** 1 Department of Neurosurgery, Tangdu Hospital, The Fourth Military Medical University, Xi’an, China; 2 Department of Neurosurgery, Xuanwu Hospital, Capital Medical University, Beijing, China; 3 Department of Interventional Neuroradiology, Beijing Neurosurgical Institute and Beijing Tiantan Hospital, Capital Medical University, Beijing, China; 4 Department of Neurosurgery, Zhujiang Hospital, Southern Medical University, Guangzhou, China; 5 Department of Neurosurgery, West China Hospital, Sichuan University, Chengdu, China

**Keywords:** Unruptured intracranial aneurysms, Natural course, Rupture rate, Predictive factor, Cohort, Protocol

## Abstract

**Background:**

Subarachnoid hemorrhage (SAH) accounts for 4.4% of cerebral vascular disease, which is one of the leading causes of death in China. Rupture of intracranial aneurysms (IAs) is the most common cause of SAH. The natural history of unruptured IAs (UIAs) and the risk factors for rupture are among the key issues regarding the pathogenesis of IA and SAH that remain unclear in the Chinese population.

**Methods:**

The China Intracranial Aneurysm Project (CIAP) is a prospective, observational, multicenter registry study of the natural courses, risk factors for the onset and rupture, treatment methods, comorbidity management and other aspects of intracranial aneurysms. To date, there are five studies in the CIAP. CIAP-1 is a prospective observational cohort study of UIAs. More than 5000 patients who will be followed for at least 1 year are expected to be enrolled in this cohort. These participants come from more than 20 centers that represent different regions in China. Enrollment began on May 1, 2017, and will take approximately 5 years. A nationwide online database of UIAs will be built. Participants’ basic, lifestyle, clinical and follow-up information will be collected. The blood samples will be stored in the Central Biological Specimen Bank. Strict standards have been established and will be followed in this study to ensure efficient implementation.

**Discussion:**

The natural course of UIAs in the Chinese population will be explored in this registry study. In addition, the risk factors for the rupture of the UIAs and the joint effect of those factors will be analyzed. The present study aims to create a nationwide database of UIAs and investigate the natural course of UIAs in China.

*Trial registration* The Natural Course of Unruptured Intracranial Aneurysms in a Chinese Cohort (ClinicalTrials.gov Identifier: NCT03117803). Registered: July 5, 2017

## Background

Cerebrovascular disease is one of the leading causes of death in China and contributes to a heavy disease burden [1]. The age-standardized prevalence, incidence, and mortality rates of stroke were 1114.8/100,000, 246.8/100,000 and 114.8/100,000 person-years, respectively, in China among those aged ≥ 20 years. Subarachnoid hemorrhage (SAH) accounts for 4.4% of cerebrovascular disease [2, 3]. Rupture of an intracranial aneurysm (IA) is the most common cause of SAH [4], which has very high morbidity and mortality [5]. A cross-sectional study of the community shows that the incidence of unruptured intracranial aneurysms (UIAs) is as high as 7% in the Chinese population of 35 to 75-year-old people [6]. Therefore, it is an urgent task to reduce the rupture risk and mortality rate.

It is critically important to clarify the natural history of UIAs and screen for risk factors for rupture. The International Study of Unruptured Intracranial Aneurysms (ISUIA) [7, 8] and Unruptured Cerebral Aneurysms Study (UCAS) [9] are the most influential studies of the natural course and annual rupture rate of UIAs. However, a relatively high degree of selectivity bias leads to lower external validity of their findings. There are still no reliable reports about the annual rupture rate of UIAs in Chinese populations.

Thus far, there is also a lack of a clear conclusion about the risk factors for rupture. Studies have shown that some factors might be related to the risk of aneurysm rupture, such as factors related to morphology (the largest diameter ≥ 7 mm, presence of ascus, etc.) [7] and pathological damage (endothelial instability, inflammatory cell infiltration, etc.). Racial factors also have a great deal of influence on the risk of aneurysm rupture. Scholars have established a “PHASES” UIA rupture prediction model based on the North American, Finnish, and Japanese populations through systematic reviews [10]. However, the results obtained by different research institutions are not consistent and might be impacted by research design and sample size. Thus, the applicability of the existing rupture model requires further verification [11].

Although a number of UIA studies have been successively carried out, the natural history of UIAs in the Chinese population is still unclear. The previous studies were mostly single-center, small-scale retrospective data analyses and cannot provide high-level research evidence. The main purposes of the present study are to calculate the annual rupture rate of UIAs in the Chinese population and identify possible risk factors for rupture.

### Study design and methods

The China Intracranial Aneurysm Project (CIAP) is a prospective, observational registry study of the natural courses, risk factors for the onset and rupture, treatment methods, comorbidity management and other aspects of IAs. To the best of the authors’ knowledge, the CIAP, sponsored by Chinese Science and Technology Ministry and National Stroke Prevention Project Committee, is the largest ongoing project evaluating IAs in China; it currently includes 5 studies. The first study in the CIAP was designed to set up and manage a UIA database of 5000 individuals and calculate the annual rate of rupture of those aneurysms. The second study aimed to identify the risk factors for aneurysm rupture and develop a multidimensional predictive model for rupture. The third study was designed to explore the strategy of antithrombotic therapy in UIA patients complicated by ischemic cardiovascular/cerebrovascular diseases. The fourth study was designed to compare the therapeutic effects of intervention surgery and craniotomy surgery in the treatment of UIAs. The fifth study was designed to develop a standardized clinical pathway for early-stage cerebral hemorrhage caused by aneurysms [12, 13].

CIAP-1, intended to establish an open research platform and a nationwide UIA cohort, is the first and most fundamental task of the entire project. It is designed to achieve the following aims:

1. Establish a nationwide public health and research platform administered by researchers, doctors, clinical center managers, Contract Research Organizations (CROs) and health management companies.
2. Establish a nationwide prospective UIA cohort as a joint effort from 20 clinical centers.
3. Clarify the natural history and annual rupture rate of UIAs in China.
4. Identify the characteristics of people with intracranial aneurysms and look for possible causes of IAs.
5. Screen for risk factors for UIA rupture and provide guidance to CIAP-2, which is designed to establish a multidimensional model to predict UIA rupture.

### Participating center qualification

The investigators will collaborate with the other 19 medical centers located in the different regions of China (Southeast, Southwest, Northwest and Northeast China). Each center has more than two referral units, has experience in the diagnosis of UIAs and has a skilled treatment team. Thus, it is believed that this cohort can adequately represent the population at the regional and national levels. During the study period, all the patients included in this study will be observed and treated in the collaborating medical centers.

### Recruitment of participants

Patients will be enrolled consecutively in all of the clinical centers and through China’s Stroke Prevention Project Committee. Patients with UIAs will be enrolled when they first visit the clinical center or are registered by the Stroke Prevention Project Committee. Patients with multiple aneurysms will also be enrolled when at least one aneurysm remains untreated. The patients will be selected according to the following selection criteria.

#### Inclusion criteria

1. Diagnosis of untreated unruptured intracranial aneurysms (by CTA, MRA or DSA).
2. Modified Rankin Scale (mRS) score of 3 or less.
3. Age older than 18 years.
4. Provision of consenting to participate in the study.

#### Exclusion criteria

1. Subarachnoid hemorrhage of unknown etiology.
2. Other cerebral arteriovenous malformations or cerebral arteriovenous fistulas.
3. Malignant tumor.
4. Fusiform aneurysms.
5. Aneurysms caused by trauma, mycotic or other factors.
6. A life expectancy of less than 1 year.
7. Participation in other clinical studies of intracranial aneurysms.
8. Refusal of follow-up.

### Outcomes

1. The primary outcome: the rupture of the aneurysm.
2. The secondary outcome: obvious changes in the morphology of the aneurysm (maximum diameter increase ≥ 1 mm or new daughter sac forms); or a new serious clinical symptom develops (such as headache).

### The plan and methods of follow-up

Every patient will be followed for at least 1 year if the aneurysm is not ruptured or treated by surgery. The Estimated Primary Completion Date is December 30, 2021. Thus, the follow-up procedure is expected to last until December 30, 2022. The participants will be contacted by phone or Internet at 3 months and 12 months and contacted face to face at 6 months. Images of computed tomography angiography (CTA)/magnetic resonance angiography (MRA)/digital subtraction angiography (DSA) and vascular ultrasound will be obtained at 6 months. The participants’ visit and evaluation schedule is shown in Table 1.

**Table 1:** Participants’ visit and evaluation schedule

Visit number	Screening period	Follow-up period		
	1	2	3	4
Research arrangement	Screening	90 ± 14 days	180 ± 30 days	365 ± 30 days
Informed consent	√			
Selection criteria	√			
Baseline information	√			
History of disease and history of medicine taking	√			
Medicine taking		√	√	√
Symptoms and physical examination	√	√	√	√
Electrocardiogram	√			
Routine blood test	√			
Blood sugar	√			
Blood lipid	√			
Homocysteine	√			
Head CT/MRI	√	√	√	√
CTA/MRA/DSA	√		√	
Vascular ultrasound	√		√	
mRS score	√	√	√	√
HAMA score	√	√	√	√
End point evaluation		√	√	√
Adverse event evaluation		√	√	√

Participants should visit at least once a year after 1 year

*CT* computed tomography, *MRI* magnetic resonance imaging, *CTA* CT angiography, *MRA* magnetic resonance angiography, *DSA* digital subtraction angiography, *mRS score* modified Rankin scale score, *HAMA score* Hamilton anxiety score

### Collection and management of data

The basic information (name, sex, age, address, etc.), lifestyle information (smoking, sleeping, exercise, job, stress, etc.), clinical data (symptoms; signs; medical history of hypertension, diabetes, cardiovascular disease, and coagulation dysfunction; family history), imaging data (the number, location, size, and morphology of aneurysms as shown on medical imaging) and follow-up information (changes in images, symptoms, and living styles; drugs taken during follow-up; etc.) will be collected during the study.

An electronic data capture (EDC) system (http://www.ciastudy.com.cn) was constructed for this project. Data are collected in each center, recorded on Case Report Forms (CRFs) and then uploaded to the EDC system.

The CRF is divided into 2 books: CRF-A is the enrollment book, while CRF-B is the follow-up book. When the CRF-A is finished, its integrity and accuracy will be verified in a timely manner by a clinical research coordinator (CRC). Then, the CRF-A will be transported to the Information Managing Center (IMC) in Beijing and uploaded by a professional database administrator (DBA) into the EDC System. A double check of the data will be implemented by the EDC system. CRF-B will be transported to the IMC 1 year later, when the follow-up has been completed. The double-check process will also be implemented for the CRF-B. The CRFs will be transported back to and stored at the enrollment center when the data upload is completed and will be stored for at least 5 years after the end of the project.

Every researcher has an account with the EDC system, which has different permissions according to the researcher’s role. The CRC can scan the data into the EDC system but cannot modify it without the permission of the manager of the IMC. Every change in the patient’s information in the database will be recorded by the system. The CIAP-1 database will be maintained for at least 5 years after the end of this study. The EDC system will continue running for other studies in the CIAP.

The DICOM data of computed tomography (CT), magnetic resonance imaging (MRI), and DSA collected by the participating centers will be transported and stored at the Medical Image Analysis Center (MIAC) in Xuanwu Hospital. These DICOM data will be reanalyzed to achieve a unified interpretation to ensure the homogeneity of the data (Table 2)

**Table 2:** The 20 participating centers in CIAP-1

Center no.	Registry number of clinical trial organizations	Name of research centers
XW01	400688385	Xuanwu Hospital, Capital Medical University
TT02	400686339	Beijing Neurosurgery Institute
ZJ03	725627505	Zhujiang Hospital, Southern Medical University
TD04	000000000	Tangdu Hospital, The Fourth Military Medical University
HX05	450756139	West China Hospital, Sichuan University
CQ06	450401805	The First Affiliated Hospital of Chongqing Medical University
FE07	401700390	The Second Hospital of Hebei Medical University
SY08	466002729	The First Affiliated Hospital of Soochow University
HH09	40135431X	Tianjin Huanhu Hospital
WY10	470005922	The First Affiliated Hospital of Wenzhou Medical University
XJ11	457601551	The First Affiliated Hospital of Xinjiang Medical University
LN12	410581610	The First Hospital of China Medical University
XY13	444885559	The Second Xiangya Hospital of Central South University
HN14	428201452	Hainan Provincial People’s Hospital
TJ15	441437486	Tongji Hospital of Tongji Medical College of Huazhong University of Science and Technology
LC16	49502066X	Liaocheng People’s Hospital
QH17	440001154	Qinghai Provincial People’s Hospital
ZS18	426600687	Zhongshan Hospital, Xiamen University
RJ19	425026572	Renji Hospital, Shanghai Jiaotong University School of Medicine
SJ20	401751630	Shijiazhuang First Hospital

20 medical centers which represent different regions in China participate in this study

.

### Collection and management of specimens

The blood samples of the patients will be collected at their first visit, transported by cold chain and stored in the Biological Specimen Bank (BSB) at Xuanwu Hospital. Every specimen has a form attached to record the information pertaining to collecting, transporting, storing and using the specimens. The form is transported and stored together with the specimen. The remaining specimens will be destroyed in the BSB at Xuanwu Hospital when the CIAP has been completed.

Some specimens and DICOM data will be transported to Tiantan Hospital to be tested and analyzed when CIAP-2 is being carried out.

### Measures to reduce bias

*Control of selection bias* This is a nationwide population-based study. Twenty centers selected from different regions of China are involved in this study. The status of diagnosis and treatment in different regions of China will be investigated in this study and will be involved in the final analysis. In addition, the patients in each center will be screened consecutively according to the selection criteria.

*Control of information bias* Blinding is being adopted in this study. The clinical research associates (CRAs) will be blinded to the study design. The statistical analysis will also be conducted blindly.

A standardized training program will be created and implemented to ensure that all of the work is undertaken by well-trained CRAs and CRCs.

The double-check process will be implemented in the process of data collection. The quality of the data will be evaluated again before analysis. Unqualified data will be corrected if possible or will be removed if the information about its accuracy is not available. The impact of missing information will be fully considered and will be subject to special analysis.

Regularly communicating with the patients will help improve the quality of the follow-up. The CRAs will call the patients on a regular basis. Furthermore, mobile applications, such as Wechat (an instant messaging and social media application), will be used to establish effective communication channels. In the case of a nonresponding patient, the CRA will try to contact the patient again several days later.

*Control of confounding factors* As many potential confounding factors as possible will be identified. Considering that this is an observational study, overly strict selection criteria are not used.

The design and implementation of this study are guided by statistical experts. Statistical methods such as standardized rate, stratified analysis, multivariate logistic regression analysis and propensity score matching will be adopted to identify the influential variables. These influential variables will be taken into consideration when calculating the annual rupture rate and when establishing the multifactor analysis model in CIAP-2.

Subgroup analyses and a case–control study design will be used when calculating the rupture rate in specific populations. The selection criteria for the exposure group and the control group will be set reasonably, and multiple groups of controls will be used if necessary.

### Monitoring of this project

A professional Project Management Committee (PMC) has been established in the CIAP, in which experts in statistics, neurosurgery, database management, and ethics are enrolled [14]. An inspection team has been established by the members of the PMC to ensure the smooth implementation of the research. The team will visit each clinical center regularly to promote the progress of this project. The first inspection will be conducted as soon as possible after screening the first patient. A CRO will be an internal part of this project to ensure the high quality of the data. The database and research materials will be censored on a regular basis by the CRO to ensure the authenticity, objectivity, accuracy and completeness of the data.

### Statistical methods

#### Sample size calculation

The sample size is calculated based on the following formula:

$$ n = \frac{{u_{\alpha }^{2} p\left( {1 - p} \right)}}{{d^{2} }} $$

The annual rupture rate is expected to be 1% [9], the allowable error is d = 0.6%, the tests will be two-sided, with an α level of 0.05. It is calculated that 1173 subjects need to be enrolled in 1 year, and 4696 subjects are needed in 4 years. The expected drop-out rate is 5%. Drop-out is identified as enrolled patients who cannot be included in the primary analysis because, for example, they did not complete the follow-up in time or they lack key information.

### Statistical analysis

- Statistical description of the clinical data.
  Continuous variables are expressed as the mean ± standard deviation and compared by one-way analysis of variance. Categorical variables are expressed as the quantity (rate) and compared with the Chi-square test. Continuous variables that do not conform to a normal distribution are expressed using the median and interquartile range and are compared by the Mann–Whitney nonparametric test. P < 0.05 is considered significant.
- Annual rupture rate calculation.
  The overall annual rupture rate of UIAs is the proportion of aneurysms that ruptured in one year, which will be calculated with the data from the 1-year assessment. When analyzing the rate in a specific population, a subgroup analysis will be performed.
- Assessment of influential factors.
  Multivariate regression analysis will be used to calculate the OR of high-risk factors for the development and rupture of aneurysms. The 95% confidence interval will also be calculated. The receiver operating characteristic curve (ROC) will be used to determine the cutoff value with the best sensitivity and specificity for the prediction of rupture.

### Ethics and dissemination

This research was approved by the Biological and Medical Ethics Committee of Tangdu Hospital (NO. 201704-13). It was also approved by the Ethics Committee of every participating center in this study. Every adverse event will be reported to the Ethics Committee in a timely manner. Every revision of the research protocol will be reported to the Ethics Committee. The investigators will strictly follow the precepts of the Declaration of Helsinki and Human Biomedical Research Ethical Issues when conducting this study. Patient information will be kept confidential throughout the study and the publication process.

## Discussion

An intracranial aneurysm is an abnormal bulging in the wall of the intracranial artery and is the most common cause of subarachnoid hemorrhage. Subarachnoid hemorrhage, mostly caused by the rupture of an UIA, is the third most common acute cerebrovascular disease, and the associated death and disability rate can be as high as 64%. It is an urgent task to control the risk factors and reduce the rupture rate of UIAs. It is equally important to reveal the natural history of UIAs and identify the risk factors for rupture.

It has been reported by UCAS that the natural course of unruptured cerebral aneurysms varies according to the size, location, and shape of the aneurysm. The annual rupture rate of UIAs is 0.95% (95% confidence interval 0.79–1.15). Larger aneurysms and those located in the posterior and anterior communicating arteries or that have a daughter sac are more likely to rupture [9]. The ISUIA also showed that the location and size of the aneurysm are predictors of rupture. Aneurysms located in the posterior circulation and posterior communicating artery rupture more easily than those located in the internal carotid artery, anterior communicating, anterior cerebral artery or middle cerebral artery. The 5-year cumulative rupture rates of aneurysms larger than 25 mm are as high as 40–50%. The 5-year cumulative rupture rates of aneurysms smaller than 7 mm are as low as 0–2.5% [7, 8].

Although some risk factors for rupture have been identified in these two large studies, the influence of other factors, such as drug history, disease history, genetics, lifestyle and stress from life and work, remain unclear. To date, the causes underlying the development of aneurysms are equally unclear. In addition, there are no reports about the rupture rate of UIAs in the Chinese population. The present study will describe the natural history of UIAs, calculate the annual rupture rate of the entire cohort as well as specific subgroups of patients, and identify the specific subgroups that have higher risks of rupture. The conclusions will provide evidence to guide the appropriate management of UIAs and develop a more reasonable UIA prevention and control strategy in China.

An observational design was chosen for this study, in which the diagnosis and treatment of disease will not be affected. Compared with an interventional study, more patients with different characteristics can be enrolled in a real-world study that has lower internal validity but superior external validity. The results of the real-world study better represent the current status of the diagnosis and treatment of diseases. At the same time, the study can be implemented for a long period of time, include a large sample size, and provide benefits for less of an investment. Furthermore, the conclusions drawn from the study might be more easily extrapolated to and more useful in clinical practice. Lastly, registration studies are safer for the participants [15, 16]. Despite the shortcomings [17], a real-world study is still the most suitable design for this study.

**Fig. 1:**
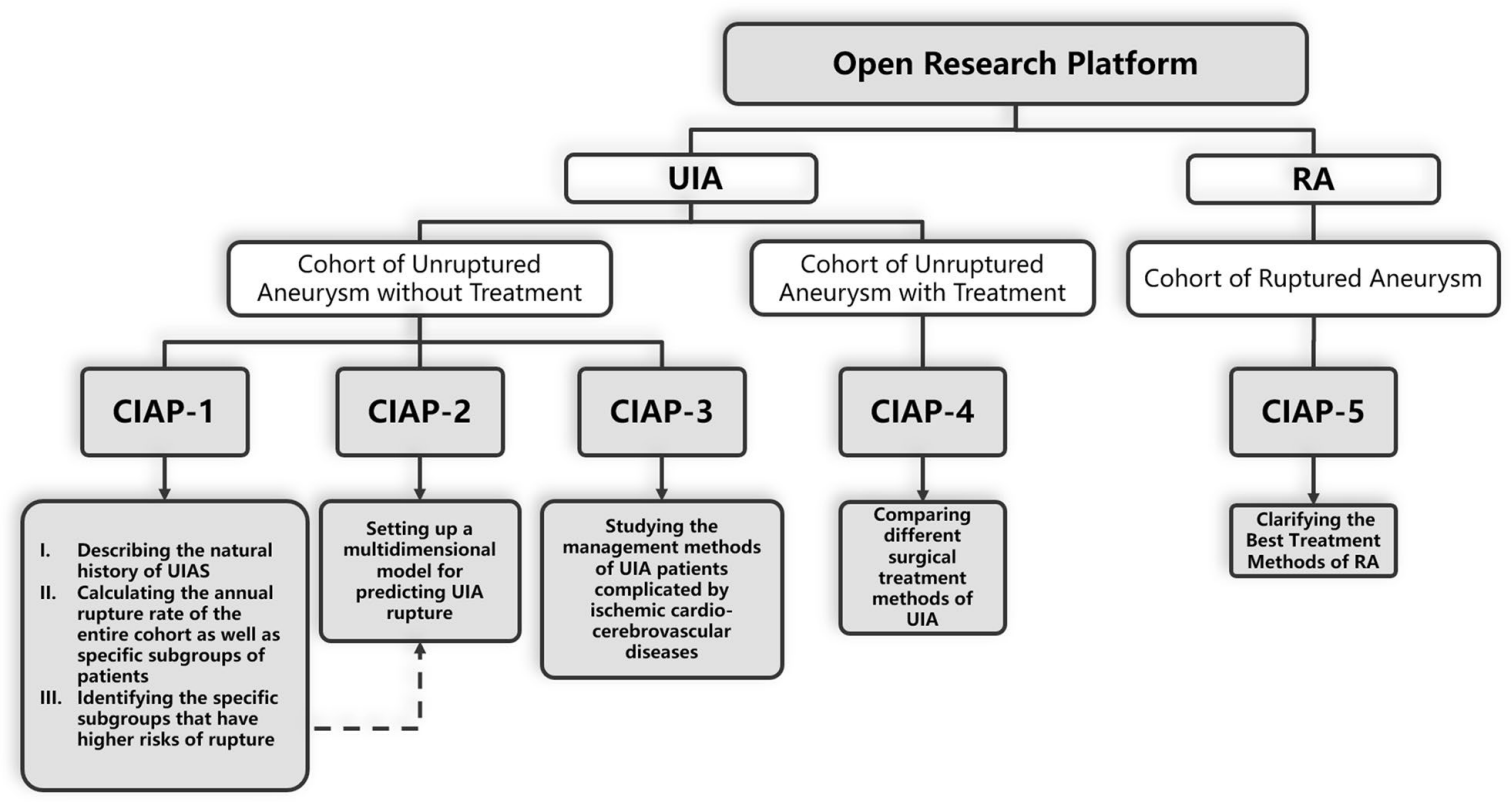
Flow chart of the CIAP. The relationships among the five studies in CIAP are described in this figure. *UIA* unruptured intracranial aneurysm, *RA* ruptured aneurysm

**Fig. 2:**
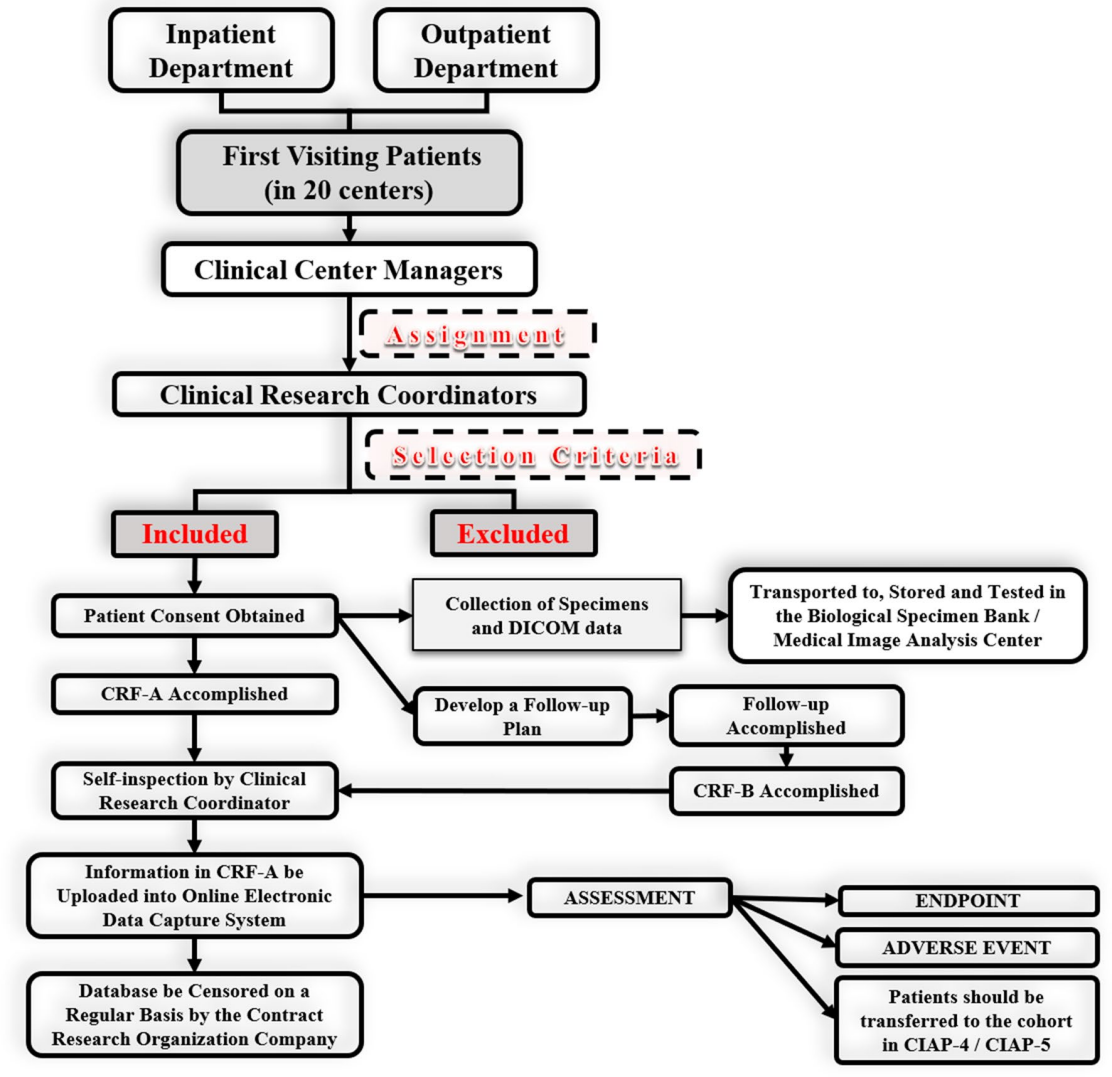
Flow chart of CIA-1. *CRF* case report form

The UIA research platform will be established in this study based on multicenter cooperation and will take advantage of the EDC system. There are 3 cohorts in this platform: the Cohort of Unruptured Aneurysm without Treatment (CIAP-1, CIAP-2, CIAP-3), Cohort of Unruptured Aneurysm with Treatment (CIAP-4), and Cohort of Ruptured Aneurysm (CIAP-5). The relationships among these five studies have been clearly described in the protocol of CIAP-2 and CIAP-4 [12, 13]. The patients in this study (CIAP-1) will be transferred to the cohort in CIAP-5 if the IA ruptures. They will be transferred to the cohort in CIAP-4 if the IA is surgically treated. Under these conditions, observation will be discontinued for this study. The patients in CIAP-1 will also be enrolled in CIAP-2/CIAP-3 if they meet the additional selection criteria (Figs. 1, 2).

Taking advantage of the EDC system, it is possible to upload, browse and examine the data at the same time in different centers. The data can be centrally managed for consistent quality control. Every change in the patient’s information in the database will be recorded by the system, which will increase the reliability and credibility of the data.

Several measures have been taken to ensure that this nationwide large-scale multicenter project is implemented adequately according to the unique standards: (1) the EDC system has an instant error reporting function; (2) self-inspection is conducted in every clinical center on a monthly basis; (3) regular training is provided to the researchers; (4) a semiannual meeting is held to summarize the research and solve any problems in the study; (5) regular censoring is conducted by the CRO; and (6) the statistical experts will follow-up with this program and evaluate the credibility and effectiveness of the data.

In addition, this project establishes a Project Management Committee that is responsible for project design and implementation, summary and analysis of the data, and writing the research report. The following institutions are also established: (1) The Central Biological Specimen Bank and Central Laboratory is established to store, test and manage the specimens. Gene and biomarker detection are conducted in the central laboratory following unified standards. (2) The Medical Image Analysis Center is established to analyse, store and manage the DICOM data. A uniformed interpretation standard of medical imaging data is made by the MIAC. (3) The Information Managing Center is responsible for the construction and maintenance of the EDC system and ensuring the security, completeness and accuracy of the data.

Limitations also exist in this study. All of the participants are Chinese; thus, the conclusions can only represent the characteristics of the Chinese population. The credibility will be impaired if the conclusions are extrapolated to other races.

In addition, information asymmetry exists. For example, regular medical examinations are not widely implemented in some remote areas. In some remote areas, patients cannot arrive at the hospital in time when the aneurysm ruptures. The incidence rate of these occurrences remains unclear. For these populations, the rupture rate might be miscalculated in this study.

Moreover, there is a serious over-treatment phenomenon in the treatment of UIAs in China, which might affect the accuracy and authenticity of our conclusions about the rupture rate. From another perspective, this study will provide solid evidence that can be used to control the overtreatment phenomenon in China.

In conclusion, this is the first nationwide study aimed at creating a database of UIAs and describing the natural course of UIAs in China. It will provide evidence to guide the choice of the most appropriate management for specific UIA patients with different risk factors.

## Data Availability

Not applicable. This manuscript does not contain any data.

## Abbreviations

BSB: Biological Specimen Bank
CIAP: China Intracranial Aneurysm Project
CRA: clinical research associate
CRC: clinical research coordinator
CRF: case report form
CRO: Contract Research Organization
CT: computed tomography
CTA: computed tomography angiography
DBA: database administrator
DSA: digital subtraction angiography
EDC: electronic data capture
IA: intracranial aneurysm
IMC: Information Managing Center
ISUIA: International Study of Unruptured Intracranial Aneurysms
MIAC: Medical Image Analysis Center
MRA: magnetic resonance angiography
MRI: magnetic resonance imaging
PMC: Project Management Committee
ROC: receiver operating characteristic curve
SAH: subarachnoid hemorrhage
UCAS: Unruptured Cerebral Aneurysms Study
UIA: unruptured IA
